# COVID-19 Paradox: The Role of Privacy Concerns and Ad Intrusiveness on Consumer’s Attitude Toward App Usage Behavior

**DOI:** 10.3389/fpsyg.2022.836060

**Published:** 2022-05-30

**Authors:** Sobia Bano, Usama Sarfraz, Anas A. Salameh, Amin Jan

**Affiliations:** ^1^Department of Management Sciences, GIFT Business School, GIFT University, Gujranwala, Pakistan; ^2^Department of Management Information Systems, College of Business Administration, Prince Sattam Bin Abdulaziz University, Al-Kharj, Saudi Arabia; ^3^Faculty of Hospitality Tourism and Wellness, Universiti Malaysia Kelantan, Kota Bharu, Malaysia

**Keywords:** pandemic, social media apps, social distancing, mobile app, online interactions

## Abstract

The COVID-19 pandemic has changed lives in an unprecedented way. The most notable and urgent requirement to combat the epidemic was to transform the way human interacts with each other. The adherence to maintaining social distance has given an upsurge to the increased usage of mobile app users. This change in human interaction for fulling their basic to social to work needs through the intervention of app usage has led to privacy concerns by users. By keeping in view the changing dynamics of the way society works, this study is an endeavor to investigate gender differences of ad intrusive and privacy concerns on app usage behavior. Employing a quantitative research design, 371 respondents were surveyed using through an online structured questionnaire. Data were analyzed by using partial least square structural equation modeling (PLS-SEM). Results suggest that advertising intrusiveness and privacy concerns are significant in determining the consumer’s attitude toward App usage, and a positive attitude toward App usage results in App usage behavior. However, gender’s moderating role in attitude toward app usage and app usage behavior is insignificant for this study. The study provides a more comprehensive understanding and complements prior insights on ads intrusiveness and privacy concerns toward app usage.

## Introduction

The global crisis of the COVID-19 epidemic has impacted the world in an unprecedented way, igniting anxiety, distress, and uncertainty among billions of people (Islam et al., 2021). To prevent the spread of this pandemic, governments across the globe restricted social life (Kim, 2020; Sardjono et al., 2021) through stay-at-home orders and closures of non-essential locations (Rippé et al., 2021). The restriction was imposed from large gatherings to the closure of educational institutes to a temporary shutdown of the economy. On the other hand, all these changes have given an upsurge to the use of technology in all fields, with few exceptions.

With technological advancement, an evident shift from computers to mobile phone usage is witnessed (Jozani et al., 2020). Around the globe and in Asia, the use of smartphones during the COVID-19 pandemic grew by 70% in 1 year (Watson, 2020). This number is the highest growth among all other digital devices. Smartphones are supported by mobile Apps, where android users can download from 3.48 million while IOS users can download from 2.22 million available apps (Ceci, 2021).

With the rise in smartphone users, the mobile advertising market is also witnessing a significant boom as marketers consider smartphones a powerful communication tool (Wang et al., 2020). According to Flood (2021), digital ad spending is expected to surge by 29.1% while standing at $491 billion spending’s this year. By 2022, it is expected that mobile’s share from total media advertising will rise to 41.3% (Wang et al., 2020). With the growth in ad spending, the frequency of ads shown to users is also increasing, and this high frequency of ads is perceived as intruding ads. The ads being perceived as intruding are likely to create negative feelings about ads which impacts the effectiveness of advertising. So, it is vital to investigate the intruding nature of ads on an individual’s attitude toward App usage.

These mobile Apps facilitate people on the one hand, but on the other hand, it is also arising concerns among people for many reasons. First mobile Apps have inbuilt sensors that collect the personal data of consumers. However, companies claim to use these data to improve users’ experience and send personal advertisements (Gal-Or et al., 2018). However, major tech joints, including Facebook, LinkedIn, and Yahoo, were exposed to data breaches of billions of users. The reportedly sharing of app users’ data to third parties is the second reason for growing privacy concerns as seven out of ten smartphone apps share data with third parties (Vallina-Rodriguez and Sundaresan, 2017). This collection and processing of personal data raise issues related to legal, social, political, and ethical issues in this era of information (Ozdemir et al., 2017; Kaushik et al., 2018).

Furthermore, this information sharing commonly results in issues related to violations of privacy while using various mobile Apps and networking sites (Crossler and Bélanger, 2019; Jozani et al., 2020). Referring to Pakistan, women are nearly half of the total population. Due to cultural limitations, the number of privacy concerns is high for women as they are exposed to harassment and cyberbullying. Moreover, since the apps have access to galleries, personal data are also prone to be breached, which may have the impact of different intensities according to gender. So, it is vital to study the role of gender in the context of app usage behavior.

The global crisis continues today, and its impact is evident in all spheres of life. Keeping in view the current dynamics of the pandemic, it is vital for companies to understand consumer concerns toward mobile app usage to maintain their competitive advantage. Since the mobile apps are designed to improve the user experience and speed up the interaction process, this study aims to investigate these issues understanding how users perceive apps, how consumers will react to privacy, and advertising intrusiveness-related issues will guide companies to enhance the consumer experience.

This study contributes by examining the factors associated with formulating the attitude of individuals and its resultant impact on app usage behavior in an Asian collectivist country’s context where technology adoption and app usage is witnessing a significant boom. Moreover, this paper also contributes to investigate the moderating effect of gender in a developing country context.

The remaining part of the paper includes a discussion related to the literature review about privacy concerns, ads intrusiveness, and attitude toward app usage, app usage behavior, and factors associated with Gender in Pakistan. Based on the literature, hypotheses are proposed. The following section deal with research methods and analysis of results. The last section includes findings and implications of those findings, limitations, and future research directions.

## Literature Review and Hypothesis Development

### Consume Privacy Concerns

Information privacy refers to the transfer of information while addressing fundamental questions of what, by whom, why, and how the information of an individual is recorded and utilized (Bandara et al., 2020). Privacy concerns reflect consumers’ worries regarding the use of information by organizations for purposes other than stated or consumers’ intentions. These privacy concerns are rising for the reason that organizations are using a range of tools to gather a large amount of user data (Bandara et al., 2020), and these data are used for commercial purposes (Jozani et al., 2020). The important thing about data is that 90% of data are generated after 2016, and 50% of these data are produced by Mobile and Internet of Things (IoT) devices (Marr, 2018). Since these devices lack security protocols, so these devices are mainly involved in privacy breaches and violations (Pour et al., 2019). The critical development in data collection is that it is no longer dependent on direct interactions as third-party data collection and secondary data usage have seen a significant rise (King and Forder, 2016). Moreover, consolidation of this data from multiple sources is compiled and resold in the market (Flyverbom et al., 2019). These activities raise privacy concerns as consumers are unaware of the process and nature of data collected (West, 2019).

Referring to privacy concerns in the Mobile Apps context, device-generated data supplements the disclosure of consumer’s information which includes but is not limited to device ID, the user’s location, contacts, and gallery (Crossler and Bélanger, 2019). Upon granting permission to apps, this kind of data is automatically shared with app owners (Dogruel et al., 2017). Moreover, these apps have the capacity to track the activities of mobile phone users (Wottrich et al., 2018). Based on these data, extensive consumers profiles are generated (King and Forder, 2016), and consumers either do not have access to these profiles or are unaware of these profiles. The commercialization of these data, which enhances the effectiveness of marketers’ efforts, raises concerns among users.

These threats are likely to result in protective behaviors, where users will adopt practices either to maintain their anonymity by using different tools (Lwin et al., 2016). Consumers adapt to these practices to keep their attitude positive toward app usage. However, when consumers feel that App owners require unnecessary or sensitive information, they may think of withdrawing the transaction (Choi et al., 2018). This shows the negative attitude toward app usage. Moreover, consumer privacy concerns have the potential to negatively influence the individual’s attitude toward mobile advertising (Martin, 2018). Based on this discussion, researchers posit the following hypothesis.

*H1*: Privacy concerns significantly impact users’ attitudes toward App usage.

### Advertising Intrusiveness

Intrusiveness is termed as a reaction that strengthens the feelings of annoyance or irritation (Hairong et al., 2002; Smink et al., 2020). When the ads disrupt the consumers’ thoughts, it is perceived as an intruding ad. Moreover, when a consumer sees an ad as more personal, it is likely to generate feelings of intrusiveness (Van Doorn and Hoekstra, 2013). The feelings of intrusiveness result in inducing negative emotions of avoidance or reactance (Baek and Morimoto, 2012; Smink et al., 2020). According to reactance theory, when consumers lack freedom or control, they are more prone to resist the message (Brehm, 1966). The consumers’ likelihood of resistance will result in negatively responding toward the source or brand (Baek and Morimoto, 2012; Ozcelik and Varnali, 2019). In the native advertising context, (Lee et al., 2016) confirmed that attitude toward non-intrusiveness results in a positive attitude and enhanced intentions of sharing. In the app’s context, Park et al. (2020) confirmed that ads in apps create feelings of intrusiveness through strength feelings vary between native and non-native app ads. Less intruding app ads result in more positive attitudinal and behavioral responses. This negative behavior will have an impact on the individual’s attitude toward app usage, failing the marketers’ efforts. Based on this discussion, the following hypothesis is proposed.

*H2*: Advertising intrusiveness negatively influences individuals’ attitudes toward App usage.

### Attitude Toward App Usage and App Usage Behavior

Attitude is defined as “the degree to which a person has a favorable or unfavorable evaluation or appraisal of behavior” (Ajzen, 1991). From this definition, it can be implied that attitude can be considered an evaluative reaction in developing a particular action, and this action can vary between favorable and unfavorable responses (Belanche et al., 2020). For this study, attitude is a general evaluation that a consumer develops while using apps. However, the nature of evaluation may vary from positive to harmful or smaller to more significant. Furthermore, attitudes are usually developed over time by frequent learning stages, and hence when an individual makes a decision, it is usually guided by the pre-formed attitude (Fazio, 1995; Belanche et al., 2020). The ease of app usage process generates more inclination of consumers to use technology (Laforet and Li, 2005). Moreover, it is also argued that perceived extrinsic and intrinsic benefits result in the usage of new technologies (Kim et al., 2016).

In the Technology Acceptance Model (TAM) replication, attitude is treated as a mediating factor (Vahdat et al., 2021). According to the study of Rivera et al. (2015), attitude mediates between technology acceptance experience and intention to use, while Vahdat et al. (2021) noted the mediating role of attitude between perceived usefulness and ease of use, with the intention to purchase. Moreover, the study by Hsu and Lin (2016) suggests that attitude mediates the relationship between perceived value and its effect on stickiness, and stickiness is an important element between the relationship of attitude toward app use and intention to purchase (Kim et al., 2015). Based on this discussion, the following hypothesis is proposed.

*H3*: Attitude toward app usage significantly influences app usage behavior.

### Role of Gender

The role of gender has gotten researchers’ significant attention in the context of digital usage, digital adoption, and marketing, which is evident from multiple research studies. For example, referring to Facebook usage, the study by Chandiramani and Sharma (2018); Kimpton et al. (2019) found the difference in intensity of Facebook usage among men and women. Moreover, women’s use of Facebook on mobile phones correlates with the increase in mobile phone usage (Van der Schyff et al., 2020). Both studies by Chandiramani and Sharma (2018) and Kimpton et al. (2019) show the high level of Facebook usage by women compared to men. In the Marketing context, the moderating role of gender has also been extensively investigated. Yuan et al. (2016) investigated gender’s moderating role in banking apps context for the relationship between perceived risk and continuance intention. The study by Joshua and Koshy (1970) concludes that men have a more positive attitude to use banking apps than women. Yu (2012) also found that men have a more substantial effect on the relationship between performance expectancy and perceived financial cost toward behavioral intentions. Evident from the literature, gender has not been studied in moderating role between attitude toward app usage and app usage behavior.

*H4*: Relationship between attitude toward app usage and App usage behavior varies among the gender.

*H5*: Gender moderates the relationship between attitude toward App usage and App usage behavior.

## Theoretical Framework

Multiple models have been used to study the factors that influence consumers’ attitudes and intentions to use technology, but the Theory of Reasoned Actions (TRA) and Technology Acceptance Model (TAM) provide a theoretical base for this paper. Both theories propose a typical pattern in which factors related to mentality have influence over intentions while factors related to expectations that impact particular behaviors (Rahi and Abd Ghani, 2016). According to TRA, consumer behavior is influenced by attitudes and intentions, which itself are determined by beliefs, and a series of studies support it (Legris et al., 2003; Wang et al., 2020). On the other hand, TAM is more applicable in the technology context (Yang, 2015), stemming from TRA but differs for attitudinal determinants (Wang et al., 2020). TAM is based on the fundamental beliefs of perceived usefulness and perceived ease of use (Davis, 1989). Perceived ease of use refers to the amount of effort required to benefit from the technology, where intruding ads enhance the degree of effort to use mobile apps.

**Figure 1:**
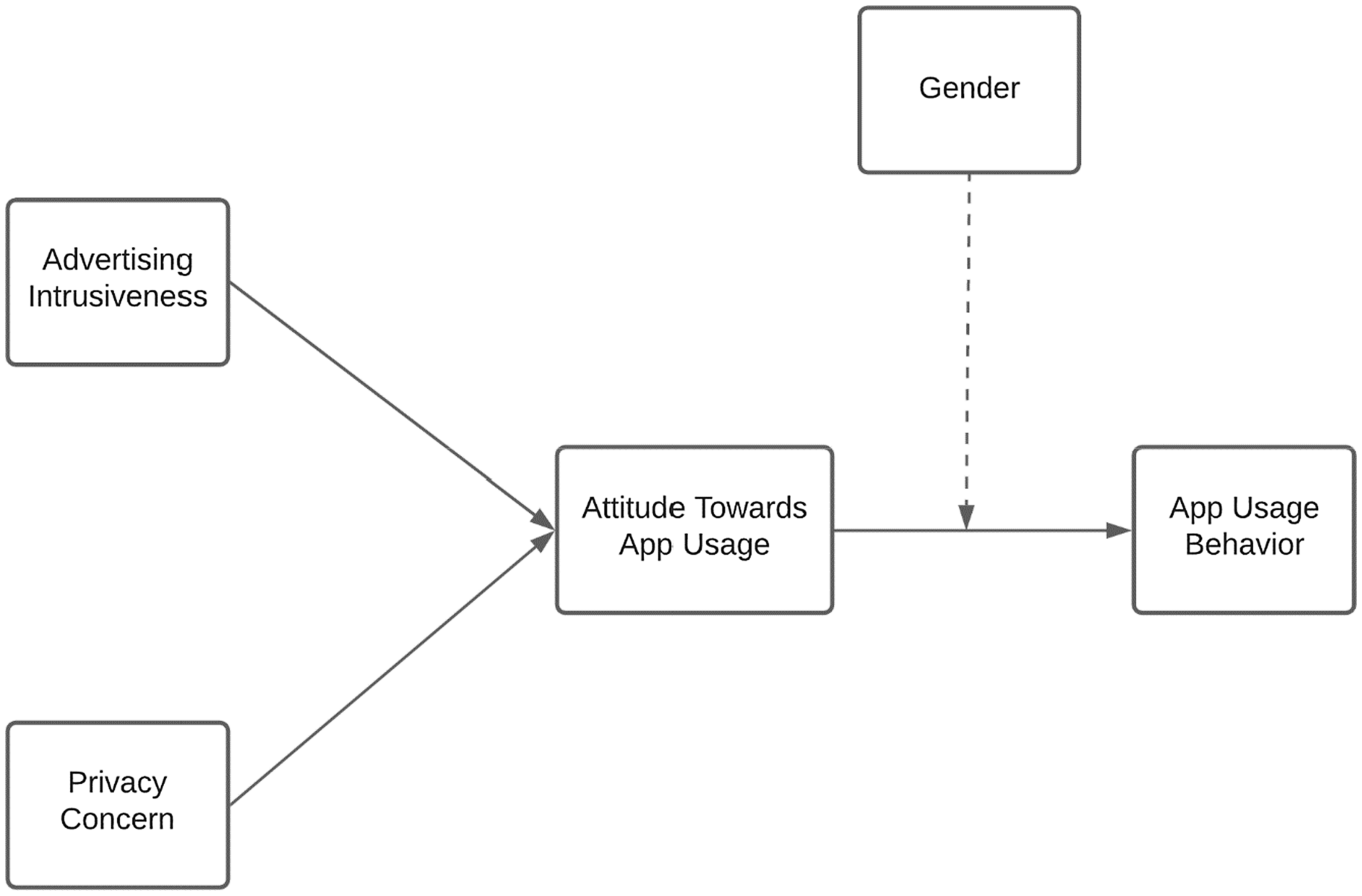
The framework of the study.

The application of TRA and TAM in mobile apps and mobile advertising is evident from the work of multiple researchers (Legris et al., 2003; Wang et al., 2020). For example, Cho et al. (2020) used TAM to investigate factors affecting consumers’ intentions in health and fitness apps’ context, and Vahdat et al. (2021) used TAM in shopping *via* mobile applications context. In addition, Shemesh and Barnoy (2020) also used this to measure intentions for health applications while used in mobile advertising’s context (Figure 1).

## Materials and Methods

### Data Collection

The data were collected through a questionnaire by sharing a link to Google Forms. The link to the questionnaire was shared through online forums. A brief information about the research objective was shared at the beginning of the survey and informed consent was obtained. The first section of the questionnaire consisted of demographic factors, including age, gender, and education, and screening questions regarding the ownership of smartphones and usage of mobile phones. This section did not include identifiable information, such as name, email, or contact number, to make people comfortable sharing the information. The screening questions were included to ensure that respondents were true representatives. The second section included statements of different scales to measure the multiple variables of the study. There was a total of twenty-one statements in the second part measured on a scale of 1–5, where one represented “strongly disagree” and five represented “strongly agree.”

Ethical considerations were ensured while conducting this study. This study did not include any financial reward for the participants, and they were informed about the non-commercial use and maintaining confidentiality and anonymity of users.

### Measurement Model

Structural equation modeling (SEM) is used to test this model. The constructs were derived from literature and were measured on a 1–5 Likert scale. Seven items scale for advertising intrusiveness was adopted from the study of Hairong et al. (2002). For privacy concerns, a six-item scale was adopted from the study of Malhotra et al. (2004). A three-item scale to measure attitude toward app usage was adopted from the study of Harrison (2015). App usage behavior scale contains five items, and it was adapted from the study of Zolkepli and Kamarulzaman (2015), and it was used by Zolkepli et al. (2021).

### Data Collection

The convenient sampling technique, which is a non-probability sampling, was employed in this study considering the time and cost effect. Qualtrics.com’s online calculator was used to determine the sample size. The online survey yielded 371 responses. After eliminating responses in which users claimed either do not own a smartphone or do not use apps on smartphones, 360 responses were selected for the final analysis. Screening questions were used to determine whether respondents should proceed to the next section or not.

### Data Analysis

Partial least square structural equation modeling (PLS-SEM) was used for data analysis. The reason for choosing SEM is that SEM has the capability to analyze the relationship between independent and dependent variables simultaneously (Hair et al., 2019). The other reason is that SEM has been widely used in exploratory research and deals well with prediction models (Hair et al., 2019). Last but not least is that irrespective of sample size, it is appropriate for non-normal data (Bandara et al., 2020), hence addressing the normality issues of data. Therefore, non-parametric bootstrapping for 5,000 subsamples was used for estimations (Hair et al., 2021). SmartPLS 3.2, a computer software, was used to conduct the data analysis (Hair et al., 2019). The analytical procedure consisted of two stages, in line with the recommendations of Hair et al. (2019). In the first stage, the reliability and validity of the measurement model were tested. In the second stage, the structural model was analyzed to test the hypothesized relationships (Sarstedt et al., 2017; Ramayah et al., 2018).

## Results

### Demographics

There were 371 respondents in the study, comprising 179 males and 192 females. This distribution is close to the natural representation of gender distribution in Pakistan’s population. 76% of respondents are between the age of 16–24, and 21% of respondents are between the ages of 25–37. This shows that data are more skewed toward young people, the right target audience, as young people are the right users of smartphones and smartphones apps. Moreover, around 98% of respondents claimed to have an education of more than 12 years, which shows that audience was educated enough to have an understanding of mobile apps usage in a non-English speaking country’s context (Table 1).

**Table 1:** Descriptive statistics.

Baseline characteristics	*n*	%	Smart phone user		Mobile app users	
			Yes	No	Yes	No
**Gender**						
Male	179	48.2	178	1	177	1
Female	192	51.8	187	5	183	4
Other						
**Age (Years)**						
0–15	2	0.5				
16–24	283	76.3				
25–37	79	21.3				
38–57	7	1.9				
**Educational qualification**						
Matric or less	9	2.4				
Intermediate (12 years)	67	18.1				
Graduate (14 years)	131	35.3				
Graduate honors (16 years)	125	33.7				
Postgraduate (>16 years)	39	10.5				

### Measurement Model

#### Convergent Validity

The measurement model is assessed by including two types of validity: convergent and discriminant. To measure convergent validity, loadings, average variance extracted, and composite reliability are used (Ramayah et al., 2018; Memon et al., 2019). One way to assess the convergent validity is using outer loading values. The threshold for outer loadings is greater than or equal to 0.7, which is the convergent validity of constructs (Chin, 2010; Hair et al., 2021); however, results are still acceptable at 0.6. All the constructs fell above the minimum threshold of 0.7 except for two items of privacy concerns, which are slightly lower than the threshold value while significantly higher than the acceptable threshold value. This means that at least 50% of variations is shared by the construct. Cronbach’s *α* was used to measure the internal consistency, and all the constructs scored higher than the minimum threshold of 0.7, established in the literature (Chin, 2010; Ramayah et al., 2018; Tikno, 2018). This shows that constructs used in this study are reliable.

Moreover, composite reliability and AVE values were also significantly higher than the minimum acceptable threshold values of 0.7 and 0.5, respectively, which is defined in the literature (Chin, 2010; Mirza et al., 2021). The results indicate that a significant amount of variance is explained by each factor, while confirming internal consistency and hence satisfying the convergent validity (Table 2).

**Table 2:** Convergent validity.

	Outer loadings	Cronbach’s *α*	Composite reliability	AVE
**Ad intrusiveness**		0.936	0.948	0.724
When the ad was shown on, I thought it was distracting.	0.828			
When the ad was shown, I thought it was disturbing.	0.855			
When the ad was shown, I thought it was forced.	0.859			
When the ad was shown, I thought it was interfering.	0.862			
When the ad was shown, I thought it was intrusive.	0.88			
When the ad was shown, I thought it was invasive.	0.852			
When the ad was shown, I thought it was obtrusive.	0.817			
**App usage behavior**		0.857	0.898	0.637
My mobile apps usage has substantially changed my life.	0.761			
I intend to use more mobile apps in the near time.	0.76			
I would use mobile apps without hesitation to satisfy my needs.	0.83			
Using mobile apps make my life easier.	0.819			
I receive a lot of benefits from my mobile apps.	0.819			
**Attitude toward App**		0.825	0.896	0.741
Overall I find using mobile apps positive.	0.857			
Overall I feel favorable toward mobile apps.	0.86			
Overall I am satisfied with mobile apps provided by my smartphone.	0.865			
**Moderating 1**	0.992	1	1	1
**Gender**	1	1	1	1
**Privacy concerns**		0.85	0.888	0.571
All things considered, the apps would cause serious privacy problems.	0.694			
Compared to others, I am more sensitive about the way mobile apps handle my personal information.	0.75			
To me, it is the most important thing to keep my privacy intact from mobile apps.	0.788			
I believe other people are too much concerned with online privacy issues.	0.677			
Compared with other subjects on my mind, personal privacy is very important.	0.813			
I am concerned about threats to my personal privacy today.	0.803			

#### Discriminant Validity

Discriminant validity is the measure of uniqueness as it tells the degree to which constructs are distinct from each other. For this study, heterotrait–monotrait (HTMT) criterion is used. The reason for choosing this is the proven efficiency of HTMT over traditional criterions (Henseler et al., 2015; Mirza et al., 2021). The respectable value to establish discriminate validity is less than 0.85 (Henseler et al., 2015; Ramayah et al., 2018); however, it is still acceptable at less than 0.9. All the items scored less than 0.85 except for attitude toward app usage, which is slightly higher than the respectable value of 0.85 but significantly lower than 0.9.

As this measurement model exhibits discriminant validity and convergent validity, so it allows proceeding with the hypotheses testing (Table 3).

**Table 3:** Discriminant validity.

	Ad Intru.	App Usg Beh	Att. App^*^Gen	Att App Usg	Gender	Priv Conc
Ad Intru.	0.85					
App Usg Beh	0.37	0.798				
Att. App Usg ^*^Gen	−0.024	0.155	1			
Att App Usg	0.404	0.703	0.143	0.861		
Gender	−0.055	−0.094	0.003	−0.102	1	
Priv Conc	0.542	0.48	0.034	0.477	−0.105	0.756

Correlation Threshold is below 0.85. Ad Intrusiveness (Ad Intru.), App Usage Behavior (App Usg Beh), Attitude toward App Usage (Att App Usg), Privacy Concerns (Priv Conc). This symbol * is auto-generated in the Smart PLS (software used for data analysis). It indicated the moderating relationship between Attitude towards app usage and gender.

* indicates the moderating relationship between Attitude towards app usage and gender.

### Structural Model

For evaluating the structural model, R2, Beta Value, and *t*-value are used, which are obtained by the bootstrapping procedure of 5,000 resamples (Hair et al., 2021). In addition, following the recommendation of Kaufmann and Gaeckler (2015) to report effect size (*f*^2^) is reported, which provides information about the substantive effect of the independent variable on the dependent variable (Hair et al., 2021).

*R*^2^ is used to assess the predictive power of the study. The value of *R*^2^ is 0.257 for attitude toward app usage, while it is 0.498 for app usage behavior. Referring to effect size, privacy concerns’ effect on attitude toward app usage is 0.127, which shows a moderate effect, while for ad intrusiveness of attitude toward app usage is small with the value of 0.04. On the other hand, for attitude toward app usage effect on app usage behavior, it is very strong with the value of 0.927. However, values suggest no effect for both gender and its moderating impact on app usage behavior.

#### Hypothesis Testing

Referring to hypothesis testing, the first hypothesis was proposed that privacy concerns influence individuals’ attitudes toward app usage. This hypothesis is confirmed as Privacy concerns (*β* = 0.365, *p <* 0.01*, t*-value *=* 5.371) are significantly positively related to attitude toward app usage. This can be interpreted as privacy shapes individuals’ attitude toward app usage in Pakistan’s context and consumers are aware of privacy-related issues. The second hypothesis proposed that ad intrusiveness influences the attitude toward app usage. This hypothesis is also confirmed as Ad intrusiveness (*β* = 0.206, *p <* 0.01*, t*-value = 3.263) had a significant effect on attitude toward app usage. This can be interpreted as ads intrusiveness shapes users’ attitude toward app usage. The third hypothesis proposed that attitude toward app usage results in app usage behavior, and the results (*β* = 0.693, *p >* 0.01*, t*-value = 18.071) confirm this hypothesis; as a result, this hypothesis is accepted. Finally, the fourth and fifth hypotheses, which were related to genders’ influence on app usage behavior, were rejected based on the results for H4 (*β* = −0.023, *p <* 0.01*, t*-value = 0.628) and H5 (*β* = 0.056, *p >* 0.01*, t*-value = 1.26), respectively. This can be interpreted as the people of Pakistan’s attitude toward app uniformly shapes app usage behavior without any gender influence (Table 4).

**Table 4:** Hypotheses testing results.

		Std Beta	ST DEV	*t*-value	*p*-value	*F* ^2^	*R* ^2^	Decision
H1	Priv Conc→Att App Usg	0.365	0.068	5.371	0	0.127	0.257	Supported
H2	Ad Intru→Att App Usg	0.206	0.063	3.263	0.001	0.04		Supported
H3	Att App Usg→App Usg Beh	0.693	0.038	18.071	0	0.927	0.498	Supported
H4	Gender→App Usg Beh	−0.023	0.037	0.628	0.53	0.001		Rejected
H5	Att. App^*^Gender→App Usg Beh	0.056	0.045	1.26	0.208	0.006		Rejected

## Discussion

The study contributes to the body of literature to investigate the App usage behavior by combining the TRA and TAM and taking Pakistan as its empirical context, where the COVID-19 pandemic has made mobile apps ubiquitous in everyday life. The confined home-based life of pandemic has accelerated the demand for mobile apps (Watson, 2020), and it motived the companies to launch new apps, as evident from increased number of apps on app stores (Ceci, 2021), to meet the evolving need of the people. This new normal has created opportunities for government, businesses & policymakers to reach a large audience through mobile apps.

The COVID-19 new variant is witnessed in every wave; it is more likely that more mobile apps will emerge as adjacent and alternatives to the traditional way of being. Keeping in view this dynamic situation, it is the right time to coordinate efforts for app developers, enterprises, government authorities, policymakers, and users. On the one hand, it supports governments in controlling the epidemic spread as these mobile apps facilitate the users in managing their day-to-day activities from basic groceries to designer wear, from socializing to attending work meetings. However, on the other hand, these mobile apps come within significant drawbacks, such as privacy concerns and ad intrusiveness, as discussed in this paper.

The results of the study depict that privacy concerns and ad intrusiveness form user attitudes toward app usage. Privacy concerns and ad intrusiveness forming attitudes are in line with the findings of different studies in the technology context (Anic et al., 2019; Wiese et al., 2020). This attitude toward app usage led to app user behavior. This is in line with the findings of past studies in the technology context (Roy, 2017; Wang et al., 2020; Vahdat et al., 2021). Further, as per the results, these two antecedents are of equal concern by both genders. This study’s results are in line with the prior work and provide theoretical support to the TRA and TAM.

In this rapidly evolving, privacy concerns and advertising intrusiveness are becoming primary concerns for marketers to address. Consumers’ privacy concerns can be reasoned with digital scandals and significant data breaches from major giants like Facebook, LinkedIn, and yahoo in the recent time period (Dastane, 2020). Moreover, since mobile phone usage is highly personal and more interactive, high privacy concerns should be expected. For this study, privacy concerns have a more substantial effect on attitude toward app usage as compared to advertising intrusiveness, as shown in Table 4.

The insights of the study have rich implications for practice as well. The app developers who are planning to develop or introduce new features in their app must work toward privacy concerns. As shared by O'Loughlin et al. (2019), improving data security by app developers will not only facilitate users, it will equally benefit the manufactures. Uptakes of the mobile app will be escalated if users are more confident about the information they share, as privacy concern is the strongest factor in forming an attitude toward app usage evident from Table 4. Currently, app developers are meeting the minimum standards, but to nurture and sustain the competitive advantage, it is essential to facilitate consumers by enhancing the security and privacy controls.

For marketers and companies utilizing a mobile app to reach a more considerable audience, need to be very careful about the intruding nature of their campaign as the results of the study, see Table 4, and have shown that ad intrusiveness will negatively impact the user’s attitude. As suggested by Truong and Simmons (2010); Wiese et al. (2020), marketers need to be vigilant. They should not be producing intruding content as it will affect the personalized relationship user have with their mobile devices. Further, the knowledge of potential consequences and characteristics of ad intrusiveness will aid marketers in making informed campaigns to lessen the negative impact (Riedel et al., 2018). Moreover, marketers can inform users about their data security and provide users with options to prevent unauthorized access (Feng and Xie, 2019).

For different departments of governments, this study also provides guidelines. To enhance the usage of mobile apps to get government services, it provides guidelines to address privacy concerns as some renowned government institutes in Pakistan, including National Databased Registration Authority (NADRA) and Federal Investigation Agency (FIA), were exposed to data breaches of citizens. Considering privacy concerns while developing apps to facilitate citizens will enhance the effectiveness of communication results and will also help in combating pandemics or disasters in a better way.

The findings also provide guidelines to policymakers and regulatory authorities, who legislate to boost the digitization process. For example, to increase smartphone penetration, regulatory bodies should ensure the privacy of consumers from app developers and operating software providers.

## Conclusion

When it comes to privacy concerns, it varies according to the context, and in mobile application’s contexts, it is still in its early stages. As optimal usage of mobile apps require users to give access to personal data, which results in privacy concern, and hence making it a notable context to study the privacy. Moreover, prior research also suggests that advertising through mobile phone apps is a new marketing practice, which is resulting in enhanced frequency of advertising messages and pushing app owners to introduce new ad placement options. This results in feelings of being irritated, which have impact on attitude toward app usage and hence requires to investigate it in mobile app’s context.

The study provides a framework assessing the antecedents of user attitude toward app usage behavior through the lens of the TAM Model and TRA. Regarding the antecedents, this study investigated the two independent variables, privacy concerns and ad intrusiveness on users’ attitudes toward app usage (H_1_ and H_2,_ respectively). The both hypotheses were significant making these important factors to be considered by app developers to have the positive app usage attitude. These two antecedents explained 26% variations in attitude toward app usage. Third hypothesis that attitude toward app usage results in app usage behavior was also significant with 50% explanation of variations in the model. Moreover, we added the moderating effect of gender on app usage behavior; however, it was insignificant which implies that privacy concerns and advertising intrusiveness are equally considered by both genders.

In simple words, the results indicate that ad intrusiveness and privacy concerns were significant and shaped users’ behavior toward app usage. Further, results revealed that no difference among different genders was found; this suggests that both genders are equally concerned about their privacy and are annoyed by ad intrusiveness.

## Limitations and Future Directions

Despite its significant findings, this study had several limitations that led to promising research opportunities. First, the study incorporates only two antecedents; future studies should integrate others antecedents like app rating. Secondly, this study studied gender differences only. It is recommended that future researchers should incorporate other demographic factors like education and age, and professions, as differences may exist in terms of privacy concerns and ad intrusiveness. Thirdly, the current study is based on general mobile app users. For future studies, it is recommended to do focused product or service research like online banking, as the privacy concerns and ad intrusiveness concerns will be different in each product and service category. Lastly, the generalizability of our conclusion is context-based; thus, future researchers can do comparative studies for more generalized results.

## Data Availability Statement

The raw data supporting the conclusions of this article will be made available by the authors, without undue reservation.

## Ethics Statement

Ethical review and approval was not required for the study on human participants in accordance with the local legislation and institutional requirements. The patients/participants provided their written informed consent to participate in this study.

## Author Contributions

All authors listed have made a substantial, direct, and intellectual contribution to the work and approved it for publication.

## Conflict of Interest

The authors declare that the research was conducted in the absence of any commercial or financial relationships that could be construed as a potential conflict of interest.

## Publisher’s Note

All claims expressed in this article are solely those of the authors and do not necessarily represent those of their affiliated organizations, or those of the publisher, the editors and the reviewers. Any product that may be evaluated in this article, or claim that may be made by its manufacturer, is not guaranteed or endorsed by the publisher.
